# Adherence to physiotherapy clinical guideline acute ankle injury and determinants of adherence: a cohort study

**DOI:** 10.1186/1471-2474-8-45

**Published:** 2007-05-22

**Authors:** Philip J van der Wees, Erik JM Hendriks, Mariette J Jansen, Hans van Beers, Rob A de Bie, Joost Dekker

**Affiliations:** 1 Department of Epidemiology, Center for Evidence Based Physiotherapy and Caphri Research Institute, Maastricht University, The Netherlands; 2 Royal Dutch Society for Physical Therapy (KNGF), Amersfoort, The Netherlands; 3 Dutch Institute for Allied Health Care (NPi), Amersfoort, The Netherlands; 4 Department of Rehabilitation Medicine, EMGO Institute, VU University Medical Center, Amsterdam, The Netherlands

## Abstract

**Background:**

Clinical guidelines are considered important instruments to improve quality in health care. In physiotherapy, insight in adherence to guidelines is limited. Knowledge of adherence is important to identify barriers and to enhance implementation. Purpose of this study is to investigate the ability to adherence to recommendations of the guideline Acute ankle injury, and to identify patient characteristics that determine adherence to the guideline.

**Methods:**

Twenty-two physiotherapists collected data of 174 patients in a prospective cohort study, in which the course of treatment was systematically registered. Indicators were used to investigate adherence to recommendations. Patient characteristics were used to identify prognostic factors that may determine adherence to the guideline. Correlation between patient characteristics and adherence to outcome-indicators (treatment sessions, functioning of patient, accomplished goals) was calculated using univariate logistic regression. To calculate explained variance of combined patient characteristics, multivariate analysis was performed.

**Results:**

Adherence to individual recommendations varied from 71% to 100%. In 99 patients (57%) the physiotherapists showed adherence to all indicators. Adherence to preset maximum of six treatment sessions for patients with severe ankle injury was 81% (132 patients).

The odds to receive more than six sessions were statistically significant for three patient characteristics: females (OR:3.89; 95%CI: 1.41–10.72), recurrent sprain (OR: 6.90; 95%CI: 2.34 – 20.37), co-morbidity (OR: 25.92; 95% CI: 6.79 – 98.93). All factors together explained 40% of the variance. Inclusion of physiotherapist characteristics in the regression model showed that work-experience reduced the odds to receive more than six sessions (OR: 0.2; 95%CI: 0.06 – 0.77), and increased explained variance to 45%.

**Conclusion:**

Adherence to the clinical guideline Acute ankle sprain showed that the guideline is applicable in daily practice. Adherence to the guideline, even in a group of physiotherapists familiar with the guideline, showed possibilities for improvement. The necessity to exceed the expected number of treatment sessions may be explained by co-morbidity and recurrent sprains. It is not clear why female patients were treated with more sessions. Experience of the physiotherapist reduced the number of treatment sessions. Quality indicators may be used for audit and feedback as part of the implementation strategy.

## Background

Evidence-based clinical guidelines are considered important tools to improve quality in health care [1-3]. Clinical guidelines are systematically developed statements designed to help practitioners and patients to make decisions about appropriate health care for specific circumstances [4]. The clinical guideline Acute ankle injury was the first physiotherapy specific evidence-based guideline in the Netherlands, published in 1998 by the Royal Dutch Society for Physical Therapy [5]. Acute lateral inversion sprains of the ankle are common in sports and activities of daily living (ADL), and concern usually young, physically active individuals [6-8]. In the guideline Acute ankle injury a Function score was introduced to assist physiotherapists to determine severity of the injury and set a prognostic profile for recovery. Research by De Bie et al. showed that the Function Score can be used to distinguish light injury from severe injury [9]. When using the Function Score, separate scores for pain, dynamic stability, loading, swelling and gait are added to a maximum of 100 points. A summary of recommendations of the clinical guideline are presented in table 1.

Implementation of clinical guidelines is influenced by several factors, like the performance gap between theory and practice [10]. The process of implementation starts with dissemination of guidelines [11]. For the implementation and evaluation of clinical guidelines it is important to know whether physiotherapists are able to adhere to the recommendations of guidelines. The actual use of and adherence to clinical guidelines, can be measured by using quality indicators [3]. Quality indicators are elements of health care for which evidence or consensus exists that they are indicative for the quality of health care, and they are usually categorised in structure, process and outcome indicators [12,13]. Structure- and process-indicators are based on recommendations for practice and intervention, while outcome-indicators represent the results of care.

Insight in adherence to clinical guidelines using indicators is important to identify barriers and provide information for further implementation of the guideline [14].

Adherence to guidelines should be put in perspective to specific patient characteristics, because recommendations in guidelines are based on the 'average' patient. For clinical decision making it is important to tailor treatment to specific individual needs of the patient. It is therefore interesting to identify possible patient characteristics that influence adherence to the guideline as prognostic factors.

Objective of this study is to investigate the ability for adherence to recommendations of the clinical guideline Acute ankle injury by measuring process and outcome of the intervention with the use of indicators, and to identify patient characteristics that determine adherence to the clinical guideline.

## Methods

### Study design

A prospective cohort study was performed to measure physiotherapy care for patients with acute ankle injury. After publication of the guideline Acute ankle injury in 1998, a group of 59 physiotherapists, who commented on the draft guideline during its development, were requested to participate in the study. This resulted in the inclusion of 22 physiotherapists. Major reason for not participating was the expected lack of patients with acute ankle injury (n = 17). Ethical approval for this study was granted by Deventer Hospitals.

Average age of the physiotherapists was 38 years, including two females. The majority (n = 17; 77%) worked in primary health care in private practice, while the remainder worked in a hospital setting. More than half (n = 12; 55%) were to some extent specialised in sports physiotherapy.

The physiotherapists collected data of consecutive patients with acute ankle sprain using a specific registration form. Criterion for inclusion was an acute inversion trauma of the ankle. Criteria for exclusion were injuries older than six weeks and severe trauma (fracture).

Patients were informed by the physiotherapists about the objective of the study, and were asked to give their informed consent to use their data for the study. The participating physiotherapists were prepared for the study in two meetings, during which the guideline Acute ankle injury was discussed, and instructions were given for registration of patient data.

### Data collection

Using the registration form, data were collected about patient characteristics. Specific details about diagnosis and treatment were collected: recurrent injury, co-morbidity, duration of complaint, normal or abnormal recovery, phase of recovery, cause of abnormal recovery, indication for physiotherapy treatment, treatment goals, interventions, accomplished treatment goals, number of treatment sessions, duration of treatment (in weeks). At the beginning and at the end of treatment the Function Score was assessed. Extraction of data was done by two researchers (PW and MJ).

### Quality indicators

To measure adherence to the guideline an initial set of 15 possible indicators were identified by two researchers (EH and MJ). Based on consensus they selected four process-indicators for final inclusion in this study. These process-indicators reflect the most important recommendations in the guideline: Use of Function score at intake and end of treatment (yes/no), Measurement of the phase of recovery at intake (yes/no); Measurement of normal or abnormal recovery at intake (yes/no); Interventions used according to guideline (yes/no). Also three outcome measures were identified as outcome-indicators: Accomplished treatment goals (completely vs. partly, stabilized, worsened) at the end of treatment for each phase of recovery at intake (%); Number of treatment sessions (maximum of six sessions if Function score ≤ 40 points at 0–5 days after injury; maximum of three sessions if Function score > 40 points at 0–5 days after injury); Function score of minimal 75 points at end of treatment.

### Prognostic factors for adherence

Patient characteristics at the beginning of treatment were used to investigate correlation with adherence to the guideline. Using the International Classification of Functioning (ICF), patient characteristics can be classified in Functioning (body function, activities, participation) and Contextual factors (personal factors and environmental factors) [15]. Based on the ICF we collected 16 patient characteristics at intake: age (years), gender (female vs. male), education (high vs. low or medium), absence of work (yes vs. no), sport (yes vs. no), load in ADL (high vs. low), duration of complaint (days), recurrent injury (yes vs. no), co-morbidity (yes vs. no), delayed recovery (yes vs. no), Function score at intake (0–100), Pain at intake (0–35), Swelling at intake (0–10), Dynamic stability at intake (0–25), Loading at intake (0–20), Gait at intake (0–10).

### Data analysis

Patient characteristics were described using descriptive statistics. To determine process and outcome indicators relative frequencies, percentages and averages of the relevant components of diagnosis and treatment process were calculated.

To investigate correlations between prognostic factors (patient characteristics) and adherence to the guideline, univariate logistic regression was carried out between each prognostic (independent) variable and the three separate outcome-indicators (dependent variables) for not adhering to the guideline: > 6 treatment sessions, < 75 points on Function score, not accomplished treatment goals.

Regression coefficient (B), Standard Error of Mean (SE) and statistical significance (p-value) were calculated. Prognostic factors with p-values of <0.10 were included in a multivariate logistic regression analysis. Patients with > 40 points on the Function score within 0–5 days after injury were excluded from analysis, because their injury was considered light and required no specific treatment.

Multivariate logistic regression was carried out using the enter model. Stepwise forward selection (LR-test; *p-inclusion *< 0.05; *p-exclusion *> 0.10), and stepwise backward elimination (LR-test; *p-*inclusion <0.05; *p-*exclusion > 0.10) were used as alternate models. Odds Ratio using e^B^, 95% Confidence Interval, and *p-*value were calculated for each prognostic variable. For each outcome indicator the percentage of explained variance (R^2^) was calculated. The three models were compared to investigate consistency in statistical significance and prediction of determinants.

To correct for potential bias, relevant physiotherapist characteristics were added in the regression model: years of experience in treating ankle injuries (≥ 3 years), specialisation (sports physiotherapy, manual therapy).

## Results

### Patient characteristics

During a period of 14 months the 22 participating physiotherapists collected data of 174 patients (mean:7.6; range:1–26). In table 2 an overview of patient characteristics is given.

### Diagnosis, treatment and evaluation

In table 3 relevant variables of physiotherapy assessment, treatment and evaluation are summarized. Twenty percent of the patients had a history of recurrent ankle injury. The average Function score was 28.4 (sd:17.5) at intake, and 87.7 (sd:13.4) at the end of treatment. The average number of treatment sessions was 5.2 (sd:2.9).

### Adherence to recommendations

Table 4 shows the adherence to recommendations based on the quality indicators. Eleven patients had a light injury with a score of more than 40 points on the Function score while duration of complaints was not more than 5 days. For 8 of these patients (73%) the maximum number of treatment sessions adhered to the outcome-indicator of maximal 3 sessions. The injury of 163 patients was classified as severe (<40 points on the Function score). For 132 of these patients (81%) the maximum number of treatment sessions adhered to the outcome-indicator (max. 6 sessions).

In 99 patients (57%) the physiotherapists showed full adherence to all indicators. Adherence to process-indicators is quite higher (155 patients; 89%) than adherence to outcome-indicators (102 patients; 59%).

### Determinants for adherence

The multivariate logistic regression analysis for determinants to adherence using the enter method, is shown in table 5. Based on univariate correlations, five prognostic factors were associated with more than six treatment sessions as outcome-indicator for not adhering to the guideline (females, active in sport, high load in ADL, recurrent sprain, co-morbidity). Based on multivariate analysis, the odds to receive more than six treatment sessions were statistically significant for three characteristics of patients: females (OR: 3.89; 95%CI: 1.41 – 10.72), recurrent injury (OR: 6.90; 95%CI: 2.34 – 20.37), co-morbidity (OR: 25.92; 95%CI: 6.79 – 98.93). All five factors together explained 40% of the variance.

Two prognostic factors were individually associated with a Function score of less than 75 points at the end of treatment (lower function score at intake, more pain at intake). These factors together explained 12% of the variance, but were not statistically significant in multivariate analysis.

Using not accomplished treatment goals as outcome-indicator for adherence, only variables of patients that enrolled in treatment at phase 1 or phase 2 of recovery were associated with a *p-value *of <0.10 in univariate analysis. Multivariate analysis of patients in phase 1 and phase 2 of recovery showed explained variance of 23% and 18% respectively.

### Consistency of explained variance in different models

Stepwise forward selection and stepwise backward elimination were used as alternate models to calculate explained variance. Both models showed an explained variance of 39% for the number of treatment sessions as outcome-indicator for not adhering to the guideline. The explained variance for all three models was consistent.

### Inclusion of physiotherapist characteristics in the regression model

Based on univariate regression, experience of the physiotherapist and specialisation in manual therapy were associated with less than six treatment sessions. Inclusion of these characteristics in multivariate logistic regression showed that physiotherapists with at least three years of experience in treating ankle injury, reduced the odds to receive more than six treatment sessions (OR: 0.2; 95%CI: 0.06 – 0.77).

It still showed statistically significant results for the odds to receive more than six treatment sessions for three patient characteristics: females, recurrent injury, co-morbidity. By including the physiotherapist characteristics in the model, the explained variance increased to 45%.

## Discussion

### Adherence to the guideline

Adherence to process-indicators was very high, signifying that the group of physiotherapists was very well capable of working according to the recommendations and that the guideline is applicable in daily practice. Although all individual outcome indicators showed adherence of more than 70%, the combined adherence was lower: 57%. Still, these data show that it is possible to work according to the recommendations in the clinical guideline.

Bekkering et al. studied adherence to the physiotherapy guideline Low back pain in a randomised clinical trial. She found adherence to recommendations varying from 20.1% (number of treatment sessions) to 91.3% (provide adequate information and advice). Overall adherence was 42% for physiotherapists who participated in an active implementation strategy [16]. A prospective cohort study by Jansen et al. showed that adherence to the physiotherapy clinical guideline Osteoarthritis of hip and knee varied from 46% to 100% [17]. Two different implementation strategies (active implementation vs. dissemination) for whiplash guidelines in physiotherapy was studied by Rebbeck et al. [18]. They found statistically significant increase of knowledge and identification of recommendations in the active intervention group compared to the passive dissemination group. Grol studied adherence to clinical guidelines by family doctors and found an average overall adherence in 30 clinical guidelines of 67%, with a range from 34 to 100% [3]. Differences in study design, registration, and performance-indicators makes it difficult to compare the outcomes of these studies.

### Determinants for adherence

A limited number of patient characteristics (female, recurrent injury, co-morbidity) were identified as determinants for adherence to the guideline, also when corrected for physiotherapist characteristics. The number of treatment sessions showed a reasonable explained variance (40%) in a combination of five patient characteristics that predict lack of adherence to the guideline. Recurrent injury and co-morbidity seem logical determinants, because recovery may take more time and requires specific intervention. However, why female patients have been treated with more sessions does not seem to be related to their condition. Adherence to the guideline is compromised when the physiotherapist has reasonable arguments to diverge from the recommendations. If we consider recurrent injury or co-morbidity as reasonable arguments to use more treatment sessions, adherence to that outcome measure will increase.

Adherence to the guideline is also influenced by physiotherapist characteristics. Experience of the physiotherapist increased adherence to the number or treatment sessions, which also increased the explained variance. Unfortunately, due to lack of sufficient data, it was impossible to further investigate the interaction between patient and physiotherapist characteristics in multi-level analysis.

In multivariate analysis, decreased function of the patient at the end of treatment (<75 points on the Function score) was significantly correlated with patient characteristics at the begin of treatment. The explained variance was low (12%). This may be a logical consequence of the fact that physiotherapists will usually continue treatment until the patient is recovered. It can therefore be expected that reasons for not reaching normal function at the end of treatment may vary and show no consistency in prognostic factors. Also the Function score was designed to be used at intake to distinguish between light and severe injury. The validity of the Function score as instrument to evaluate recovery is unknown, and more research is required to investigate the evaluative use of the Function score.

### Generalisation of the outcome

Characteristics of the patients in this study were similar to data from an earlier study concerning patients with acute ankle injury [19], which showed that the group of patients in this study resembles the normal population of patients with acute ankle injury.

The physiotherapists that participated in this study, commented on the draft version of the guideline Acute ankle injury. Furthermore, the major reason for not participating was a lack of patients with acute ankle injury. That means a possible selection took place of physiotherapists who have more knowledge about the clinical guideline, who treat more patients with acute ankle injury, and who are more competent in treating ankle injuries. This may also explain that the majority of the physiotherapists were to some extend specialised in sports physiotherapy, because about half of all acute ankle injuries occur during sports activities. We should also notice that the physiotherapists were instructed to use a registration form, which may have guided them in adhering to the guideline.

Our conclusion is that adherence to recommendations in the guideline is possible, but possibly overestimates the adherence, which may reduce the external validity of this study.

### Development of indicators

Although the use of indicators to measure quality of health care and performance in health care is common, methodology to develop quality indicators is still in development [20]. Campbell et al. described research methods to develop indicators [13]. They distinguished non-systematic from systematic methods to develop indicators. Systematic development can be based on evidence e.g. derived from recommendations in clinical guidelines, and on consensus procedures. Although our methodology for development of indicators was systematic by using recommendations from an evidence-based clinical guideline, the consensus procedure was basic and may require further refinement. Delphi procedures and panel meetings may enhance the production of valid, reliable and useful indicators [12-14,21].

### Application of indicators

Data generated using quality indicators can be used for a variety of purposes. Practitioners can use them as benchmark to compare their performance with others. Indicators are also useful to develop implementation strategies for clinical guidelines [14]. From the financiers perspective, indicators can be used to reward or penalize care provision [13]. The number of treatment sessions reflects both process and outcome of care. Insurance companies may be interested in adherence to the number of treatment sessions as outcome-indicator because it directly reflects the costs of treatment. This study indicates that it is not possible to use a maximum of six treatment sessions as a fixed indicator for all acute ankle injuries, because several patient characteristics influence adherence to that specific indicator.

### Implications for implementation

Implementation of clinical guidelines requires a tailored and multi-facetted approach [1]. The results of this study show that the guideline Acute ankle sprain is applicable in daily practice, which is promising for further implementation. Although the use of the registration form as guidance or reminder for the physiotherapist may have biased the outcome of this study, it may also be an effective means for further implementation. The development of a web based electronic registration form may be helpful for further implementation of the clinical guideline. Electronic registration also enables to create benchmarks by using quality indicators for feedback and monitoring.

## Conclusion

Adherence to the clinical guideline Acute ankle sprain by a specific group of physiotherapists showed that the guideline is applicable in daily practice and the results are promising for further implementation. Adherence to the guideline, even in a specific group familiar with the guideline, showed possibilities for improvement. The necessity to exceed the expected number of treatment sessions may be explained by co-morbidity and recurrent sprains. It is not clear why female patients were treated with more sessions. Experience of the physiotherapist reduced the number of treatment sessions. Quality indicators may be used for audit and feedback as part of the implementation strategy.

## Competing interests

The author(s) declare that they have no competing interests.

## Authors' contributions

PW extracted data, carried out data analysis and drafted the manuscript. EH set up the design of the study, collected and extracted data, participated in data analysis, and selected quality indicators. MJ collected data and selected quality indicators. HB participated in data analysis. RB and JD contributed to design of the study, data analysis and critically revised the manuscript. All authors read and approved the final manuscript.

## Pre-publication history

The pre-publication history for this paper can be accessed here:



## Figures and Tables

**Table 1:** Summary of recommendations in clinical guideline Acute ankle injury

**Function Score**
If a patient scores more than 40 points within the first five days after injury, it is considered a light injury, which requires no specific physiotherapy treatment. For these patients full recovery to normal ADL activities (except sport) can be expected in 14 days, which equals a Function score of at least 75 points.
**Light injury**
For patients with light injuries and without necessity for sport-specific rehabilitation the guideline recommends a maximum of three treatment sessions.
**Severe injury**
Severe injuries may require up to six treatment sessions within six weeks. Choice of interventions is based on normal recovery after ligament sprains, going from acute inflammation to proliferation and remodelling of the injured tissue. Furthermore, the guideline recommends that exercise therapy should be the main focus of the intervention, while physical modalities like Ultrasound or Electrotherapy are not recommended to be part of the intervention.
**Sports rehabilitation**
Sport specific rehabilitation requires specific treatment, which may last for about 12 weeks. Progressive increase of loading. Exercise therapy: from static to dynamic; from single-task to multi-task; from cyclic to non-cyclic exercises

**Table 2:** Characteristics of patients (N = 174)

		**N**	**(%)**	**x**	**sd**
Gender	Male	100	(57)		
	Female	74	(43)		
Age	Years			29.8	(12.7)
Education	Low/Medium	112	(65)		
	High	61	(35)		
	Missing	1			
Participation	Employed*	91	(52)		
	Student	49	(28)		
	Housewife/-man	16	(9)		
	Disabled	3	(2)		
	Unemployed	2	(1)		
	Missing	13	(8)		
Load ADL	Low/normal	71	(41)		
	High	103	(59)		
Sports	No	66	(38)		
	Yes	108	(62)		

* 34 patients (20%) suffered during treatment from temporary absence of work

**Table 3:** Diagnosis, treatment and evaluation (n = 174)

		**N**	**(%)**	**x**	**(sd)**
Recurrent injury	No	138	(80)		
	Yes	35	(20)		
	Missing	1			
Co-morbidity	No	158	(91)		
	Yes	16	(9)		
Duration complaint	Days	174		7.1	(7.1)
Normal recovery	No	30	(17)		
	Yes	142	(82)		
	Unknown	1	(0.5)		
	Missing	1	(0.5)		
Phase of recovery	Phase 1	74	(43)		
	Phase 2	79	(45)		
	Phase 3	14	(8)		
	Phase 4	7	(4)		
Function Score	Intake (assessment)	174		28.4	(17.5)
(max of 100 points)	End (evaluation)			87.4	(13.4)
Treatment sessions	Number of sessions	174		5.2	(2.9)

**Table 4:** Results of adherence to process and outcome indicators (N = 174)

**Indicator**	**Type**	**Criterion**	**Adherence**	
			**N**	**(%)**

Function score (intake and end)	Process	Assessed	171	(98)
Recovery (phase at intake)	Process	Assessed	174	(100)
Recovery (normal/abnormal)	Process	Assessed	172	(99)
Interventions	Process	According guideline	160	(92)
Accomplished treatment goals:	Outcome	Fully accomplished		
Phase 1 (n = 73^a^)			56	(77)
Phase 2 (n = 78^a^)			66	(85)
Phase 3 (n = 14)			10	(71)
Phase 4 (n = 7)			6	(86)
# treatment sessions with severe injury Function score ≤ 40 points (N = 163)	Outcome	Maximal 6 sessions	132	(81)
# treatment sessions with light injury Function score > 40 points (N = 11)	Outcome	Maximal 3 sessions	8	(73)
Function score at end treatment	Outcome	≥ 75 points	152	(87)
Overall adherence	Outcome	Adherence to all indicators	99	(57)
		Adherence to process indicators	155	(89)
		Adherence to outcome indicators	102	(59)

^a ^one missing value

**Table 5:** Regression analysis (enter model) for prognostic factors that determine non-adherence

**Outcome-indicator (non-adherence)**	**Prognostic factors**	**OR**	**95% CI**	***p***	**R^**2 **^(%)**
Treatment sessions (> 6) (n = 163)					40
	Gender (f/m)	3.89	1.41 – 10.72	0.009	
	Sport (y/n)	2.66	0.73 – 9.68	0.14	
	Load in ADL (high/low)	1.75	0.51 – 6.02	0.38	
	Recurrent sprain (y/n)	6.90	2.34 – 20.37	< 0.001	
	Other pathology (y/n)	25.92	6.79 – 98.93	< 0.001	
Function score (< 75) (n = 163)					12
	Function score begin (0–100)	0.99	0.95 – 1.05	0.95	
	Pain (0–35)	0.81	0.67 – 0.97	0.89	
Treatment goals phase 1 not accomplished (n = 68)					23
	Function score begin (0–100)	1.01	0.86 – 1.18	0.95	
	Pain (0–35)	0.76	0.53 – 1.10	0.15	
	Dynamic stability (0–25)	0.85	0.62 – 1.16	0.30	
Treatment goals phase 2 not accomplished (n = 74)					18
	Swelling (0–10)	0.66	0.49 – 0.91	0.01	

Prognostic factors: selected for multiple regression analysis; OR: Odds Ratio using e^B^; CI: Confidence Interval; *p*: *p*-value; R^2^: Nagelkerke's percentage of explained variance
